# Determination of Volatile Compounds in Four Commercial Samples of Japanese Green Algae Using Solid Phase Microextraction Gas Chromatography Mass Spectrometry

**DOI:** 10.1155/2014/289780

**Published:** 2014-01-27

**Authors:** Masayoshi Yamamoto, Susanne Baldermann, Keisuke Yoshikawa, Akira Fujita, Nobuyuki Mase, Naoharu Watanabe

**Affiliations:** ^1^Integrated Bioscience Section, Graduate School of Science and Technology, Shizuoka University, 836 Ohya, Suruga-ku, Shizuoka 422-8529, Japan; ^2^Leibniz-Institute of Vegetable and Ornamental Crops, Großbeeren/Erfurt e.V., Theodor-Echternmeyer-Weg 1, 14979 Großbeeren, Germany; ^3^Institute of Nutritional Science, University of Potsdam, Arthur-Scheunert-Allee 114-116, 14558 Nuthetal, Germany; ^4^Technical Research Institute, R&D Center, T. Hasegawa Company Ltd., 29-7 Kariyado, Nakahara-ku, Kawasaki 211-0022, Japan; ^5^Graduate School of Engineering, Shizuoka University, 3-5-1 Johoku, Naka-ku, Hamamatsu 432-8561, Japan

## Abstract

Green algae are of great economic importance. Seaweed is consumed fresh or as seasoning in Japan. The commercial value is determined by quality, color, and flavor and is also strongly influenced by the production area. Our research, based on solid phase microextraction gas chromatography mass spectrometry (SPME-GC-MS), has revealed that volatile compounds differ intensely in the four varieties of commercial green algae. Accordingly, 41 major volatile compounds were identified. Heptadecene was the most abundant compound from Okayama (*Ulva prolifera*), Tokushima (*Ulva prolifera*), and Ehime prefecture (*Ulva linza*). Apocarotenoids, such as ionones, and their derivatives were prominent volatiles in algae from Okayama (*Ulva prolifera*) and Tokushima prefecture (*Ulva prolifera*). Volatile, short chained apocarotenoids are among the most potent flavor components and contribute to the flavor of fresh, processed algae, and algae-based products. Benzaldehyde was predominant in seaweed from Shizuoka prefecture (*Monostroma nitidum*). Multivariant statistical analysis (PCA) enabled simple discrimination of the samples based on their volatile profiles. This work shows the potential of SPME-GC-MS coupled with multivariant analysis to discriminate between samples of different geographical and botanical origins and form the basis for development of authentication methods of green algae products, including seasonings.

## 1. Introduction 

Seaweeds have been traditionally used as medicines, food, and seasoning [1, 2]. Most seaweeds are of commercial importance in foodstuffs, cosmetics, and dyes. Moreover, they serve as source for biological active compounds. Today 7.5–8 million tons of macroalgae are produced per annum and their estimated value is US$ 5.5–6 billion [3]. Seaweeds are widely distributed throughout the world and are predominantly consumed in Japan, China, Korea, South Australia, New Zealand, Polynesia, and South America [4].

Certain countries or regions have reputations for producing high-quality green algae, including Aonori (a high quality, merchandised Japanese green seaweed), and are able to demand a significantly higher price for their products. In Japan, high-value production areas include the river Shimanto in Kochi prefecture and river Yoshino in Tokushima prefecture. There is a strong interest in the authenticity of these commercial products and up to now consumers have to rely on package information to provide confirmation that the product is from a high-quality region with a traceability system, such as the Food Marketing Research and Information Center in Japan [5].

GC-MS techniques coupled with multi-variant analysis are becoming increasingly important tools to monitor the distribution of counterfeit foodstuffs and have been used in authenticating many food products, such as tea, olive oil, honey, cheese, and wine [6–12].

We newly report on nontargeted SPME-GC-MS followed by multi-variant statistical analysis in Japanese green macroalgae. Significant differences in the volatile profiles of green algae from four production areas were detected, implying that markers could potentially be identified using this analytical-statistical approach.

## 2. Experimental 

### 2.1. Commercial Dried Green Algae Samples

*Ulva prolifera, Ulva linza, *and* Monostroma nitidum*, commercially all sold as “Aonori” (Japanese green algae), were subjected to sensory evaluation and used for solid-phase microextraction (SPME) method development. *Ulva prolifera *(2 samples), *Ulva linza,* and *Monostroma nitidum* were obtained from Okayama, Tokushima, Ehime, and Shizuoka prefecture, respectively. Commercial Japanese green algae were purchased from a wholesale agency in Japan. The samples were placed in air tight plastic bags in the dark at room temperature. Prior to analysis, the samples were ground to a fine powder using liquid nitrogen.

### 2.2. Sensory Evaluation

The sensory tests were carried out by nine untrained panelists consuming regularly green algae. Hedonic sensory attributes evaluated in this study were color, taste, and overall acceptability of four green algae samples. The panelists (6 male, 3 female) were between 26- and 40-year old. A nine point hedonic scale (0–8), anchored by dislike extremely to like extremely for the overall score, light colored to strongly colored for the green color, and weak to intense for the taste, was employed. More precisely, the panelists were asked to evaluate the following aroma attributes animalic, floral, spicy, fatty, green note, marine-like, fresh (waterly), powdery, and leather-like common flavor attributes of green algae. Samples were served in glass beakers and provided randomly to panelists. Sensory evaluation procedures were explained to the panelists prior the tests.

### 2.3. Solid Phase Microextraction (SPME)

Polydimethylsiloxane fibers (100 *μ*m) were mounted in a SPME manual holder (Supelco, Bellefonte, PA, USA). All fibers were conditioned prior to analyses, according to the manufacturer's recommendations. Various parameters (extraction method, time and temperature) influencing SPME analysis were optimized. For each analysis 10 g of sample was placed in a 40 cm^3^ vial, spiked with 1 mg of the internal standard ethyldecanoate, and then sealed with an aluminum crimp cap provided with a needle-pierceable polytetrafluoroethylene/silicone septum. Each sample vial was placed at 50°C for 60 min to equilibrate and then the septum was pierced with the SPME needle. Fibers were exposed to the sample headspace for 60 min. After the extraction time, fibers were retracted into the needle, transferred immediately to the injection port of a GC, and desorbed at 230°C for 1 min.

### 2.4. GC/MS Analysis

The compounds collected in the headspace above the green algae samples were analyzed by GC connected to a mass spectrometer (MS) (Shimadzu GC-MSQP5000), which was controlled by a Class-5000 workstation. Separation was carried out on a Supelcowax 10 fused silica capillary column 30 m × 0.25 mm i.d., 0.25 *μ*m film thickness (Supelco, Bellefonte, PA, USA). Injection was in splitless mode for 1 min at 230°C with a specific SPME insert. The GC temperature was raised from 50 to 160°C at 3°C min^−1^ and from 160 to 240°C at 10°C min^−1^. The mass spectra were scanned at 70 eV over a mass range from *m/z* 50 to 280. Nonisothermal linear RI (Kovats type) was calculated after the analysis of a C11–C30 *n*-alkane series (GL Science) under the same conditions. Volatile compounds were tentatively identified by comparing their mass spectra with those of the reference database (NIST Mass Spectral Data 98′ edition). Compounds were attributed to molecules through their individual mass spectrum, retention indices (RI), and retention time (RT). In addition, pure reference compounds were used to confirm the results. Flavor properties were assigned, based on reported odor descriptions [3, 13, 14].

### 2.5. Data Analysis

The relative peak areas were calculated in relation to the peak area of the internal standard ethyldecanoate. The identified volatile compounds were classified into different groups of molecules, such as alkanes, alkenes, ketones, aldehydes, sulfur compounds, alcohols, and esters. Unidentified compounds were excluded from this analysis. The relative peak areas of the different compounds in each category were calculated as a ratio of the total peak area. Statistical analyses were conducted using XL-STAT, version 2013 (Addinsoft, New York, USA).

## 3. Results and Discussion

**Figure 1:**
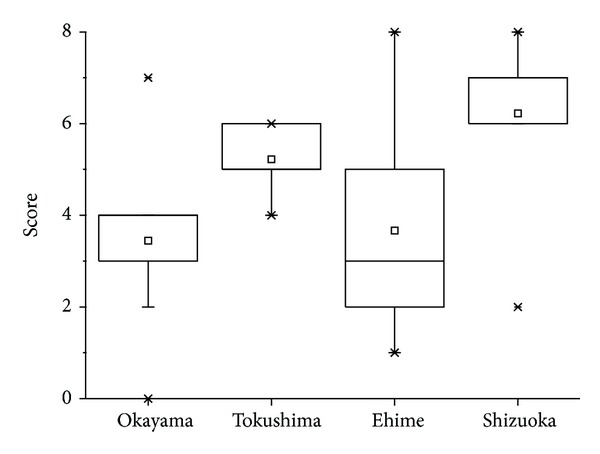
Overall scores of the sensory evaluation of green algae samples.

**Figure 2:**
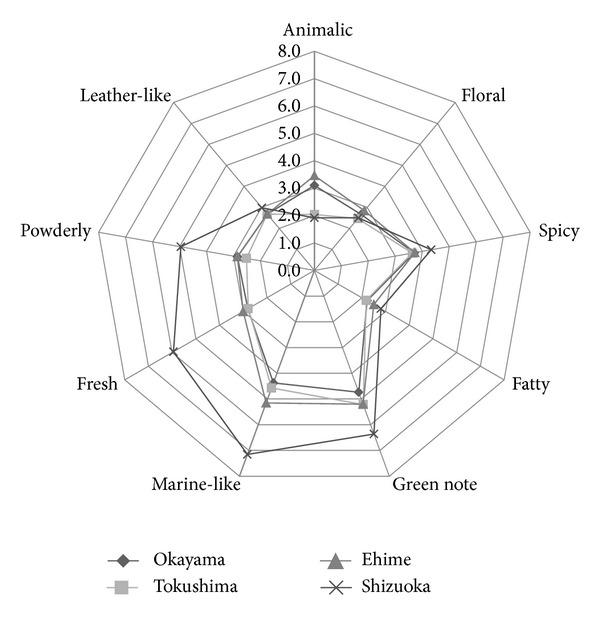
Sensory characterisation of green algae samples. The following aroma attributes were determined based on panelist's evaluation: animalic, floral, spicy, fatty, green note, marine-like, fresh (waterly), powdery, and leather-like.

The results of the sensory analysis revealed that it was impossible to differentiate between the samples based on the overall score (Figure 1). The same applies also to taste and color (data not shown). Based on the nine aroma attributes (animalic, floral, spicy, fatty, green note, marine-like, fresh (waterly), powdery, and leather-like), it was found that the samples from Okayama, Tokushima, and Ehime prefecture are uniform. However, the sample from Shizuoka prefecture was differently evaluated in respect of its green-note, marine-like, fresh, and powdery aroma (Figure 2).

As a result, it is impossible to distinguish between commercially sold “Aonori” samples based on sensory evaluation. Solely, in taxonomically different samples Okayama, Tokushima, and Ehime (*Ulva *species) *versus *Shizuoka (*Monostroma *species) somewhat different scores could be obtained.

Volatile compounds are fundamental for algae odor and aroma. Therefore, it is not surprising that volatile compounds in algae have been investigated and reports evaluating volatile metabolites, for example, in brown and red algae, are available in the current literature [15–18].

GC-MS techniques coupled with multi-variant analysis have been used in authenticating many food products and should be tested in this study for its practicability to distinguish different commercially sold Japanese green algae samples.

Forty-one major volatile compounds, classed as alkanes, alkenes, ketones, aldehydes, sulfur compounds, alcohols, and esters, were identified in the four commercial Japanese green algae (Table 1). These compounds are derived from different precursors, including the amino acids, fatty acids, and carotenoids (Table 1).

**Figure 3:**
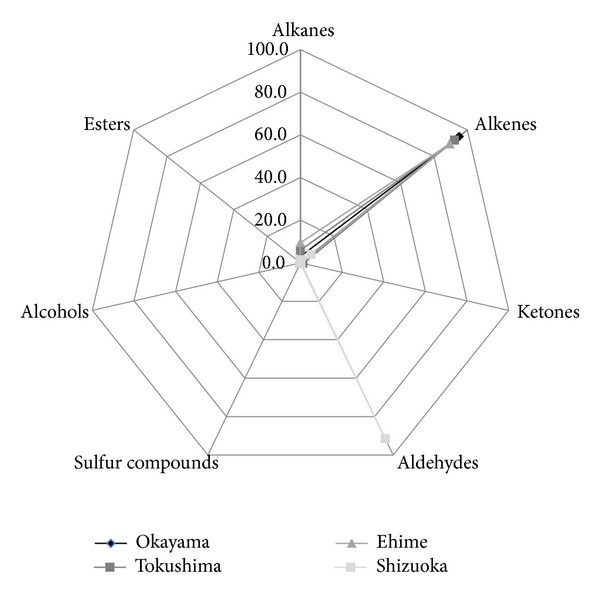
Chemical classification of volatile compounds in relative concentrations (*n* = 3) in different commercial green algae samples.

Hydrocarbons, including alkanes and alkenes (e.g., tetradecene, pentadecane, hexadecane, and octadecene), were abundant in three (Okayama, Tokushima, and Ehime) of the four samples (Figure 3).

The relative concentrations of alkenes vary among the samples from different algae of the order *Ulvales*. Green algae of the *Ulva* species are rich in alkenes: 95% in Okayama, 92.3% in Tokushima, and 89.4% in Ehime prefecture. In contrast, the green algae of the *Monostroma* species from Shizuoka prefecture contain only 6.3% of alkenes. An opposite trend has been found for the relative content of aldehydes. Very low relative contents are present in algae from Okayama (0.2%), Tokushima (0.1%), and Ehime (0.1%) and about 91.5% in algae from Shizuoka prefecture.

Heptadecanes, derived from fatty acids, were identified as major components among all samples. 7-Heptadecane has been reported to be one of the major components in marine green algae (*Ulva pertusa *and* Chara vulgaris*) and is known to be the character impact compound of edible kelp. It also contributes to the off-flavor in water [1, 19]. There is a seasonal variation in the concentration of 7-heptadecene and phytol in marine algae. It was found that 7-heptadecane was usually present in appreciable amounts throughout the year except during summer [20].

**Figure 4:**
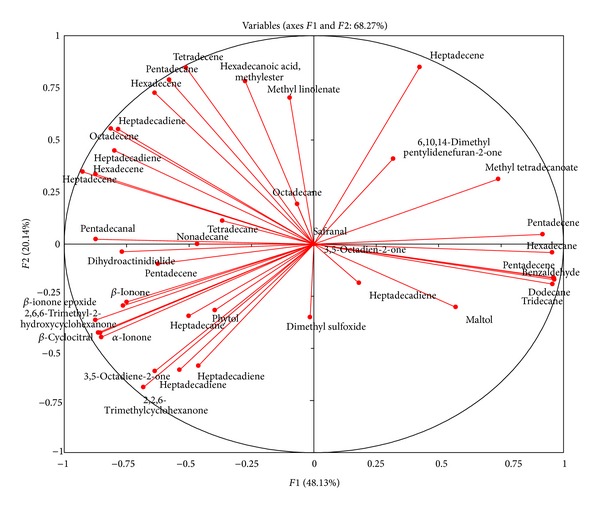
Correlation circle of volatile compounds from different green algae samples. The profiling was carried out based on 41 peaks and detected corresponding peak area ratios. Samples were independently analyzed in triplicate. The data was analyzed by PCA using XL-STAT 2013.2.

Ketones, sulfur compounds, and esters occurred in similar concentration ranges in all of the samples. Principal component analysis was performed to identify key volatiles contributing to the flavor characteristic of the four commercial algae samples.  Figure 4 shows the correlation circle of factor 1 against factor 2 and helped us to identify major volatiles contributing to the distinct flavor properties of the commercial samples.

Our analysis revealed benzaldehyde, an important compound in algae from Shizuoka prefecture, originated from the amino acid biosynthesis pathway. Benzaldehyde contributes to the sweet, almond odor of fruits and flowers and is characterized by low odor thresholds (350–3500 ppb). Benzaldehyde can also be liberated from glycosidically bound precursors in various foods. In addition, it has been reported that concentrations increase during manufacturing processes, for example, production of apple puree or roasting of sesame seeds [21].

Several hydrocarbons such as tridecane, pentadecane, hexadecane, and dodecane were identified as relevant odor compounds in seaweed from Shizuoka prefecture.

It is noticeable that the proportion of volatile apocarotenoids is remarkably high in the investigated algae varieties. The importance of carotenoid-derived flavor compounds in algae has been previously reported. Volatile apocarotenoids, such as the potent flavor compound *β*-ionone, are produced by a diversity of algae taxa, for example, *Ulothrix fimbriata* and Asakusa Nori (*Porphyra tenera*) [22, 23]. Apart from chemical formation pathways [24], it has been demonstrated that functional carotenoid-cleavage like enzymes contribute to the formation of volatile apocarotenoids in algae [25].

2,2,6-Trimethylcyclohexanone, (*E, Z*)-3,5-octadiene-2-one, 2,6,6-trimethyl-2-hydroxycyclohexa-none, *α*-ionone, *β*-ionone, and *β*-ionone epoxide are abundant in Okayama and Tokushima. Dihydroactinidiolide makes an important contribution to the volatile profile of algae from Tokushima. It is also an important component in green tea, with a sweet peach-like flavor [24]. In addition, *β*-cyclocitral and phytol are characteristic compounds influencing the flavor of algae from Okayama and Tokushima prefecture: they contribute to fresh and floral odors, respectively [18]. The ketones, such as *β*-cyclocitral, *β*-ionone, and *β*-ionone epoxide and phytol, are generated *via *degradation of carotenoids. *α*-Ionone contributes to the violet, wood odor; *β*-ionone contributes to the violet-like and characteristic seaweed odor; *β*-ionone epoxide contributes to the sweet berry odor. These are important aroma impact compounds characterized by very low odor thresholds; for example, *β*-ionone has an odor threshold of 0.007 ppm [26]. *β*-Ionone accounts for 12.5%, 15.7%, 5.9%, and 0.7% of the volatiles in the algae harvested from Okayama, Tokushima, Ehime, and Shizuoka prefecture, respectively. Moreover, thermal degradation products of *β*-carotene, including 2,2,6-trimethylcyclohexanone, benzaldehyde, 2,6,6-trimethyl-2-hydroxycyclohexanone, *β*-cyclocitral, 5,6-epoxy-*β-*ionone and dihydroactinidiolide, were previously reported [24].

After the removal of undesirable material, green algae are sun dried and packaged. It might be speculated that similar reactions occur during the sun drying processing of green algae and may have a strong impact on the quality of algae products. The occurrence of apocarotenoids is one of the most important indicators of authenticity for *Ulva prolifera, Ulva linza,* and *Monostroma nitidum*.

Another class of aroma compounds, the aliphatic acid methyl, esters, including hexadecanoic acid, methyl, ester, and methyl linolenate, was abundant in algae from Shizuoka prefecture. Methyl linolenate serves as aprecursor of (*E, Z*)-3,5-octadien-2-one and is one of the most odor-active compounds [27]. It has been found in cod liver oil and *Ulva pertusa* and contributes to the melon-like odor. In this study, (*E, Z*)-3,5-octadien-2-one was detected in all samples and was more abundant in *Ulva prolifera*.

To explain the overall characteristics of the four algae samples, further studies are necessary to elucidate the contribution of single components and identify unknown compounds.

**Figure 5:**
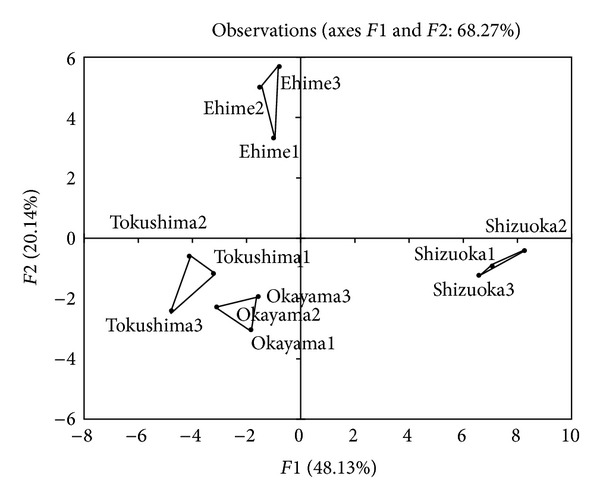
Scoring plots of nontargeted profiling of green algae samples of different origin by GC-MS-SPME. The profiling was carried out based on 41 peaks and detected corresponding peak area ratios. Samples were independently analyzed in triplicate. The data were analyzed by PCA using XL-STAT 2013.2.

Multi-variant analysis of known components was performed on the mass spectral data to explain the overall characteristics of the differences among the samples. PCA was performed using a transformed data set consisting of correlation matrix of 41 variables yielding in 11 eigenvectors. Five factors explained 92.39% of the total variance, indicating that a reduced number of volatile compounds could explain the overall characteristics of the four different samples. Factor 1 explained 48.13% of the total variance and factor 2 explained 20.14% of the total variance. Both factors together enable discrimination of the four algae samples from different biological and geographical origins (Figure 5). It is interesting that there are fewer differences in the volatile profiles of samples from close geographic origins, such as Tokushima and Okayama, compared with samples which are further away from each other, for example, Tokushima and Ehime. Future studies should be performed with a higher number of replicates and taking into account testing sets.

## 4. Conclusions

The volatile profiles of different commercial Japanese green algae samples, originating from Okayama, Tokushima, Ehime, and Shizuoka, were described for the first time. Multivariate analysis enabled discrimination of the samples, based on their botanical and geographical origin. Hitherto, the authenticity of these commercial products is based on package information and this study could form the analytical basis of authenticity studies of dried green algae samples using the SPME-GC-MS-method, followed by multivariante analysis.

## Figures and Tables

**Table 1:** Volatile compounds of different green algae samples presented as relative ratios compared to the internal standard ethyldecanoate.

No.	Volatile compounds	Formula	Class	Origin	Odor description	RI	Relative contents (% averaged GC peak area ratio (*n* = 3))							
							Okayama	STD	Tokushima	STD	Ehime	STD	Shizuoka	STD
**1**	Dodecane	C_10_H_22_	Alkane		Alkane (B)	1185	ND		ND		ND		1.2	0.0010
**2**	Tridecane	C_13_H_28_	Alkane	Fatty acids	Alkane (B)	1297	0.6	0.0003	0.6	0.0008	ND		23.2	0.0298
**3**	2,2,6-Trimethylcyclohexanone	C_9_H_16_O	Ketone	Carotenoids	Camphoraceous tobacco notes; thujone-like (A)	1308	0.7	0.0011	0.7	0.0026	ND		ND	
**4**	Tetradecane	C_14_H_30_	Alkane	Fatty acids	Alkane (B)	1397	3.9	0.0020	11.4	0.0097	7.1	0.0158	6.0	0.0065
**5**	Tetradecene	C_14_H_28_	Alkene			1444	0.5	0.0013	0.9	0.0005	2.1	0.0025	ND	
**6**	Pentadecane	C_15_H_32_	Alkene		Alkane (B)	1502	83.6	0.0622	251.1	0.1181	420.5	0.6057	ND	
**7**	(*E,E*)-3,5-Octadien-2-one	C_8_H_12_O	Ketone	Fatty acids, carotenoids	Almond (C)	1517	ND		ND		ND		ND	
**8**	Benzaldehyde	C_7_H_5_O	Aldehyde	Amino acids	Sweet, strong almond (A) almond, burnt sugar (B)	1517	1.7	0.0028	2.3	0.0068	0.9	0.0015	3777.3	7.4504
**9**	Pentadecene isomer 1	C_15_H_30_	Alkene			1523	2.4	0.0028	3.6	0.0025	4.7	0.0057	11.7	0.0326
**10**	Pentadecene isomer 2	C_15_H_30_	Alkene			1546	0.7	0.0011	2.3	0.0025	1.0	0.0010	0.5	0.0028
**11**	Dimethyl sulfoxide	C_2_H_6_OS	Sulfur compound	Amino acids		1557	8.2	0.0457	16.5	0.0944	8.0	0.0500	12.3	0.0221
**12**	(*E,Z*)-3,5-Octadien-2-one	C_8_H_12_O	Ketone	Fatty acids, carotenoids	Melon-like (C)	1567	2.7	0.0100	2.1	0.0065	0.6	0.0052	0.3	0.0029
**13**	Pentadecene isomer 3	C_15_H_30_	Alkene			1576	ND		ND		ND		5.1	0.0085
**14**	2,6,6-Trimethyl-2-hydroxycyclohexanone	C_9_H_16_O_2_	Ketone	Carotenoids	Sweet tobacco-like aroma with herbaceous undertones (A)	1594	8.6	0.0237	9.6	0.0223	4.1	0.0053	ND	
**15**	Hexadecane	C_16_H_34_	Alkane		Alkane (B)	1601	4.3	0.0089	5.6	0.0122	7.7	0.0144	28.2	0.0563
**16**	*β*-Cyclocitral	C_10_H_16_O	Aldehyde	Carotenoids	Fresh (B)	1612	3.5	0.0085	3.5	0.0074	1.4	0.0010	ND	
**17**	Hexadecene isomer 1	C_16_H_32_	Alkene			1619	1.2	0.0010	2.5	0.0027	2.1	0.0025	ND	
**18**	Hexadecene isomer 2	C_16_H_32_	Alkene			1624	0.9	0.0024	1.1	0.0026	2.2	0.0004	ND	
**19**	Safranal	C_10_H_14_O	Aldehyde	Carotenoids		1637	ND		ND		ND		ND	
**20**	Heptadecane	C_17_H_36_	Alkane		Alkane (B)	1707	56.1	0.0610	25.2	0.0936	25.5	0.2227	12.4	0.0993
**21**	7-Heptadecene	C_17_H_34_	Alkene			1722	2909.7	1.0740	4003.3	3.3306	3954.3	5.5587	155.5	0.3312
**22**	Heptadecene isomer 1	C_17_H_34_	Alkene			1733	1.8	0.0193	ND		12.0	0.0030	7.0	0.0169
**23**	Heptadecadiene isomer 1	C_17_H_32_	Alkene			1757	103.4	0.0604	139.2	0.1109	148.7	0.1725	64.3	0.1246
**24**	Heptadecadiene isomer 2	C_17_H_32_	Alkene			1766	1.0	0.0021	1.2	0.0018	0.5	0.0047	0.7	0.0015
**25**	Heptadecadiene isomer 3	C_17_H_32_	Alkene			1769	4.3	0.0022	2.2	0.0011	0.8	0.0074	0.7	0.0104
**26**	Heptadecadiene isomer 4	C_17_H_32_	Alkene			1772	3.8	0.0037	5.3	0.0036	5.9	0.0041	1.1	0.0198
**27**	Heptadecadiene isomer 5	C_17_H_32_	Alkene			1779	0.8	0.0022	ND		0.3	0.0059	0.5	0.0091
**28**	Octadecane	C_18_H_38_	Alkane		Alkane (B)	1801	3.0	0.0051	1.6	0.0042	3.2	0.0189	2.1	0.0059
**29**	Octadecene	C_18_H_36_	Alkene			1817	11.2	0.0072	12.8	0.0059	18.1	0.0085	0.9	0.0081
**30**	*α-*Ionone	C_13_H_20_O	Ketone	Carotenoids	Warm, woody, violet-floral (A)	1842	4.6	0.0039	5.6	0.0139	1.8	0.0085	ND	
**31**	Nonadecane	C_19_H_40_	Alkane		Alkane (B)	1898	2.9	0.0119	1.1	0.0039	2.0	0.0029	0.7	0.0033
**32**	*β*-*Ionone *	C_13_H_20_O	Ketone	Carotenoids	Warm, woody, dry (A)	1927	12.5	0.1087	15.7	0.0144	5.9	0.0040	0.7	0.0031
**33**	Maltol	C_6_H_6_O_3_	Ketone		Caramel-butterscotch (A) caramel (B)	1960	0.6	0.0082	ND		ND		1.0	0.0059
**34**	*β-*Ionone epoxide	C_13_H_20_O_2_	Ketone	Carotenoids	Sweet berry (A)	1980	2.1	0.0181	2.8	0.0025	0.9	0.0006	ND	
**35**	Methyl tetradecanoate	C_15_H_30_O_2_	Ester		Faint onion, honey (A), orris (A) (B)	2010	0.9	0.0051	0.0	0.0000	1.3	0.0017	1.7	0.0036
**36**	Pentadecanal	C_15_H_30_O	Aldehyde		Fresh (B)	2034	1.8	0.0103	3.1	0.0033	1.8	0.0103	ND	
**37**	6,10,14-Trimethyl-2-pentadecanone	C_18_H_36_O	Ketone	Carotenoids		2139	2.9	0.0168	6.7	0.0174	7.0	0.0160	7.4	0.0427
**38**	Hexadecanoic acid, methylester	C_17_H_34_O_2_	Ester	Fatty acids		2217	5.2	0.0210	2.2	0.0038	9.0	0.0133	2.2	0.0029
**39**	Dihydroactinidiolide	C_11_H_16_O_2_	Ketone	Carotenoids	Musky or coumarin-like (A)	2347	3.0	0.0363	8.4	0.0199	4.2	0.0022	0.4	0.0034
**40**	Methyl linolenate	C_19_H_32_O_2_	Ester	Fatty acids		2566	4.1	0.0390	2.7	0.0063	7.3	0.0268	3.4	0.0057
**41**	Phytol	C_20_H_40_O	Alcohol	Chlorophyll	Faint floral (A) flower (B)	2580	2.8	0.0191	1.2	0.0050	0.9	0.0023	0.7	0.0071

(A) Food and Agriculture Organization of the united nations (http://www.fao.org), (B) Flavornet (http://www.flavornet.org), and (C) Hu and Pan, 2000 [14].
